# Ant Species Differences Determined by Epistasis between Brood and Worker Genomes

**DOI:** 10.1371/journal.pone.0000994

**Published:** 2007-10-03

**Authors:** Timothy A. Linksvayer

**Affiliations:** Department of Biology, Indiana University, Bloomington, Indiana, United States of America; University of Edinburgh, United Kingdom

## Abstract

Epistasis arising from physiological interactions between gene products often contributes to species differences, particularly those involved in reproductive isolation. In social organisms, phenotypes are influenced by the genotypes of multiple interacting individuals. In theory, social interactions can give rise to an additional type of epistasis between the genomes of social partners that can contribute to species differences. Using a full-factorial cross-fostering design with three species of closely related *Temnothorax* ants, I found that adult worker size was determined by an interaction between the genotypes of developing brood and care-giving workers, i.e. intergenomic epistasis. Such intergenomic social epistasis provides a strong signature of coevolution between social partners. These results demonstrate that just as physiologically interacting genes coevolve, diverge, and contribute to species differences, so do socially interacting genes. Coevolution and conflict between social partners, especially relatives such as parents and offspring, has long been recognized as having widespread evolutionary effects. This coevolutionary process may often result in coevolved socially-interacting gene complexes that contribute to species differences.

## Introduction

Social interactions are ubiquitous and often strongly influence both fitness and trait expression [1]–[3]. When social interactions have a genetic basis, the social environment is heritable and a focal individual's phenotype is influenced directly by its own genotype (direct genetic effects) and indirectly by the genotypes of social partners (indirect genetic effects) [2], [4]–[7]. Interactions between genes within individuals give rise to physiological epistasis, which is central to evolutionary theories for local adaptation, population differentiation, and the evolution of species differences [8]–[13]. With physiological epistasis, a gene's effect depends on the context provided by the rest of the genome. When the effects of genes expressed in one individual depend on genes expressed in social partners, epistasis between the genomes of social partners results [14]–[17]. While such intergenomic epistasis arising from social interactions has been little studied, theory suggests it may influence many of the same evolutionary processes as physiological epistasis [14]–[17].

With physiological epistasis, phenotypes are determined by groups of interacting genes, and during the course of population differentiation, populations are expected to diverge for different coevolved gene complexes [9]–[12]. Disruption of these coevolved complexes is manifested as hybrid breakdown, the fitness decline that typically occurs following hybridization between divergent populations [9]–[12], [18]. Similarly, with intergenomic epistasis, phenotypes are determined by combinations of interacting genes expressed in different individuals. As a result, interacting social phenotypes are expected to coevolve, and divergent populations are expected to harbor distinct coevolved gene complexes [14]–[16], [19]. The coevolution and divergence of socially-interacting genes may affect a broad range of traits, including those involved in mate recognition and compatibility, social dominance, and familial interactions [20]–[26]. In some cases, the concerted evolution of socially-interacting phenotypes may result in runaway dynamics, including accelerating arms races, rapid host race formation, and speciation [14]–[16], [19].

In social insects, the environment experienced by developing brood is determined by the social milieu of the colony [27]. In particular, the nutritional environment and microclimate of developing brood is provided and actively regulated by adult sibling workers, so that workers play a fundamental role in shaping brood developmental trajectories [27], [28]. Earlier research discovered high levels of heritable variation within a population of the ant *Temnothorax curvispinosus* for direct effects, worker effects, and queen effects on female mass and reproductive caste (i.e. worker vs. queen development). This study demonstrated that the social environment provided by adult queen and worker nestmates makes substantial contributions to genetic architecture for the studied phenotypes and can be shaped by selection [29].

Here I investigate the contributions of brood genotype (direct effects) and care-giving worker genotype (worker effects) to interspecific differences in worker size for three closely related ant species in the genus *Temnothorax*; *T. ambiguus*, *T. curvispinosus*, and *T. longispinosus*. If these species have diverged for different coevolved worker-brood genotype combinations, the effects of brood and worker genotype should be context dependent, i.e. there should be evidence for intergenomic epistasis. These species have widely overlapping ranges in eastern North America and broadly similar natural histories [30], [31]. They are all generalist scavengers and nest in acorns and other preformed cavities, but there are measurable interspecific differences in worker body size [32], behavior [33], and microhabitat preference [30], [31]. Worker size affects many aspects of foraging and nesting ecology, such as the types of available food and nesting resources, and is likely shaped by selection both within and between colonies [27]. Worker size differs between sympatric pairs of ant species in *Temnothorax* as well as in the closely related genus *Leptothorax*, perhaps due to character displacement as a result of competition for food or nesting resources [32].

Just as the production of recombinants between lineages is a powerful tool to study the intragenomic basis of phenotypic differences between lineages [34], cross-fostering creates different combinations of the genotypes of focal individuals and their social partners and is a powerful tool to study the intergenomic basis of phenotypic differences between lineages [4], [35], [36]. I used a full-factorial cross-fostering design with brood (i.e. focal individuals) and care-giving workers of the three *Temnothorax* species. For each of the nine worker-brood combinations, I constructed 15 replicate experimental colonies, for a total of 135 experimental colonies, each composed of 15 workers and 25 larvae.

## Results

**Figure 1:**
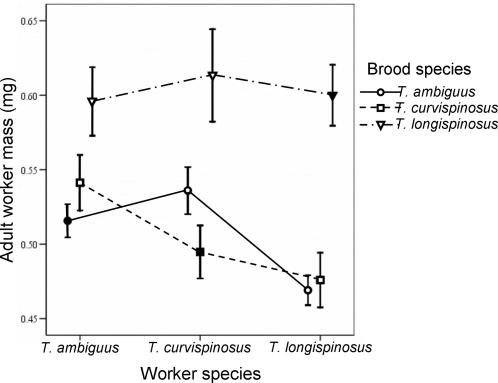
Mass of workers produced when cross-fostered brood were reared to adulthood. The adult mass of focal individuals depended on their own genotype (i.e. brood species) as well as the genotype of care-giving workers (i.e. worker species). There were main effects of brood and worker species, and a brood-by-worker interaction. Means and 95% confidence intervals are shown. Filled symbols denote conspecific worker-brood combinations and open symbols denote heterospecific worker-brood combinations. Lines connect worker-brood combinations with the same brood species and the crossing of lines indicates the brood-by-worker interaction.

All cross-fostered larvae were accepted by the groups of workers to which they were assigned. The 135 experimental colonies reared a total of 1,154 workers, as well as 149 queens and 181 males. I analyzed the main and interaction effects of brood (focal individual) species and worker species on the mass of newly emerged focal individuals. The species identity of brood influenced their adult mass as new workers (brood species main effect, mixed model, *F*_(2,107)_ = 40.54, *P*<0.0001); *T. longispinosus* individuals were larger than *T. ambiguus* or *T. curvispinosus* individuals regardless of which worker species reared them (Fig. 1). The species identity of care-giving workers also affected the mass of new workers produced (worker species main effect, mixed model, *F*_(2,118)_ = 6.60, *P* = 0.0019); individuals reared by *T. longispinosus* workers were the smallest (Fig. 1), despite the fact that care-giving *T. longispinosus* workers were the largest (see below). Finally, there was an interaction between the species identity of brood and care-giving workers for new worker mass (brood species-by-worker species interaction, mixed model, *F*_(4,105)_ = 3.19, *P* = 0.016); the size of *T. ambiguus* and *T. curvispinosus* individuals depended on which worker species reared them while the size of *T. longispinosus* individuals did not (Fig. 1). There were no detectable main or interaction effects on the mass of new queens (all *P*>0.05) and for male mass only brood species identity had an effect (brood species main effect, mixed model, *F*_(2,122)_ = 22.51, *P*<0.0001).

Some heterospecific worker-brood combinations produced new worker phenotypes that were more extreme than the corresponding conspecific worker-brood combinations (Fig. 1). For example, *T. ambiguus* brood reared by *T. longispinosus* workers developed into smaller workers than when reared by conspecific workers (Fisher's post hoc test, df = 103, *P*<0.0001), while *T. curvispinosus* brood reared by *T. ambiguus* workers were larger than those reared by conspecific workers (Fisher's post hoc test, df = 106, *P* = 0.015).

Colonies with the different worker-brood combinations produced similar low numbers of gynes (Kruskal Wallis test, χ*^2^* = 8.53, df = 8, *P* = 0.38) but different numbers of males (χ*^2^* = 29.35, df = 8, *P*<0.001) and workers (χ*^2^* = 34.06, df = 8, *P*<0.001). In particular, more males were produced in colonies with *T. longispinosus* brood (mean±s.d., 2.91±3.72) than colonies with *T. ambiguus* (0.76±0.91) or *T. curvispinosus* (0.31±0.67) brood, indicating that *T. longispinosus* broods contained relatively more haploid eggs. Mean colony worker production was affected by the species identity of brood (mean±s.d. by brood species: *T. ambiguus*, 11.49±5.73; *T. curvispinosus*, 9.16±3.53; *T. longispinsosus*, 7.16±4.00) as well as workers (mean±sd by worker species: *T. longispinosus*, 10.93±5.03; *T. ambiguus*, 9.40±5.03; *T. curvispinosus*, 7.47±5.01).

**Figure 2:**
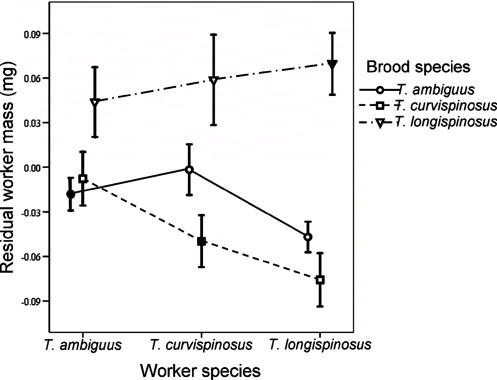
Residual mass of workers produced from cross-fostered brood after controlling for differences in colony productivity. After controlling for colony differences in the total number of individuals produced, the main effect of worker species became non-significant, suggesting that there was a tradeoff between the different worker species for the size and number of individuals reared. The main effect of brood species and the worker-by-brood interaction remained significant, and the overall pattern for residual worker mass is very similar to the pattern for mass shown in Figure 1. Means and 95% confidence intervals are shown. Filled symbols denote conspecific worker-brood combinations and open symbols denote heterospecific worker-brood combinations. Lines connect worker-brood combinations with the same brood species and the crossing of lines indicates the brood-by-worker interaction.

This variation in colony productivity potentially affected the mean mass of new workers through a size-number trade-off. Indeed, the total number of individuals produced was negatively correlated with the mean mass of new workers across colonies (ρ = −0.259, *n* = 135, *P* = 0.003). When variation in total colony productivity was controlled for (Fig. 2), the main effect of worker species on new worker mass became non-significant (mixed model, *F*_(2,124)_ = 2.56, *P* = 0.082), while the remaining effects did not change (brood species, mixed model, *F*_(2,113)_ = 36.15, *P*<0.0001; brood-by-worker species interaction, mixed model, *F*_(4,111)_ = 3.46, *P* = 0.011).

Field-collected workers used to create experimental colonies showed the expected pattern of interspecific variation in mass (ANOVA, *F*_(2,682)_ = 167.5, *P*<0.001; mean mass in mg±2 s.e.m.: *T. ambiguus*, 0.356±0.012, *n* = 164; *T. curvispinosus* 0.300±0.010, *n* = 210; *T. longispinosus* 0.440±0.011, *n* = 309). Note that new workers produced during the experiment were larger because they were reared in laboratory conditions with unlimited food (Fig. 1).

## Discussion

This study demonstrates that phenotypic differences in worker mass among *Temnothorax* ant species are determined by intrinsic differences within developing brood, but these differences also depend on the social context provided by care-giving workers. These interspecific differences could have genetic and environmental components but are likely mainly of genetic origin for several reasons. First, contributions from pre-fostering sources of environmental variation between species for initial larval size are minimal because larvae gain the vast majority of mass post-fostering [29]. Second, there is no evidence for differences in the condition of care-giving workers, as worker survival over the course of the experiment did not differ according to worker species identity (generalized linear model, *P* = 0.348). Finally, post-fostering sources of environmental variation are minimal because all colonies were kept under constant laboratory conditions.

Thus, the results indicate that phenotypic differences in worker mass between the three studied *Temnothorax* species are mainly due to direct genetic effects (i.e. brood genotype) and direct-by-indirect genetic interactions (i.e. brood-by-worker genotype interaction). This interaction is a type of genotype-by-environment interaction, in which the environment is provided to developing brood by care-giving workers [14]–[16]. Because the worker-provided social environment is heritable, the interaction is also a type of genotype-by-genotype interaction, or epistasis between genomes of developing brood and care-giving workers [14]–[16]. While intragenomic epistasis arises from the physiological interaction of gene products within an organism, possible mechanistic bases for the observed intergenomic epistasis include behavioral interactions governing larval solicitation and worker provisioning, pheromonal signaling and response, and differential physiological responses of brood genotypes to the developmental environments provided by workers [15], [16], [26], [37], [38]. The three species displayed two different patterns of intergenomic epistasis; *T. longispinosus* focal individuals developed into similarly-sized new workers regardless of the rearing environment, whereas the mass of new *T. ambiguus* and *T. curvispinosus* workers depended on the rearing environment (Fig. 1), suggesting that development is more canalized in *T. longispinosus* than in the other two species.

There was also evidence for a context-independent effect of worker genotype, but this effect became non-significant after controlling for colony differences in the total number of individuals produced, suggesting that the worker effect was mainly associated with a size-number tradeoff (Fig. 2). That is, on average the different worker species reared either relatively few large workers or more but smaller workers.

Phenotypes of social organisms are determined by combinations of direct and indirect genetic effects expressed in focal individuals and their social partners, respectively. When certain combinations are favored, theory predicts these effects will coevolve, and the concerted coevolution of interacting social phenotypes will lead to the buildup of direct-indirect genetic correlations within populations and distinct coevolved direct-indirect gene combinations in divergent lineages [14]–[16], [19], [26]. In accordance with these predictions, in a previous within-population study of *T. curvispinosus* there were strong direct-indirect genetic correlations for female mass and caste [29]. Furthermore, the worker-brood intergenomic epistasis detected in the current study is a strong signature that the three *Temnothorax* species harbor distinct coevolved direct-indirect gene complexes. In some cases, heterospecific worker-brood combinations produced new worker phenotypes that were more extreme than the corresponding conspecific worker-brood combinations (Fig. 1). If the mass of new workers produced by conspecific brood-worker combinations is near a fitness peak, the heterospecific combinations that are more extreme may be associated with decreased fitness. Thus, cross-fostering may have broken up coevolved worker-brood gene combinations, just as crossing divergent lineages tends to disrupt coevolved physiological gene combinations [9]–[12], [18]. Altogether, these results suggest that the studied ant phenotypes are shaped by the coevolution of direct and indirect effects expressed in focal individuals and their nestmates. Prolonged and intimate social interactions among queen, worker, and brood nestmates are a fundamental characteristic of social insect colonies [27], guaranteeing that this coevolutionary process has the potential to influence all aspects of social insect evolution [28], [29].

Female reproductive caste, a phenotype central to the origin and elaboration of eusociality, is especially likely to be subject to the coevolution of direct and indirect effects because caste development depends on brood responses to the nutritional environment provided by adult nestmates [28], [29], [39], [40]. In the current study, all worker-brood combinations produced similar low numbers of gynes, probably because the colonies were relatively small. Other social insect studies involving taxonomically more extreme cross-fostering have demonstrated that caste phenotypes are readily disrupted [41], [42].

The non-additive interactions between workers and brood described here are similar to theorized non-additive, synergistic interactions between social partners that have been incorporated into models for the evolution of social behavior [43]–[45] as extensions of Hamilton's Rule [46]–[48]. These non-additive social interactions all potentially give rise to epistasis for fitness and affect the evolutionary dynamics of nestmate phenotypes. Worker-brood epistasis involves a special type of social interaction, though, because the social partners are at different life history stages, and these interactions, like parent-offspring interactions [14], [15], [19], shape the developmental trajectory of brood.

Interacting nestmates may often have differing interests regarding their own and their nestmates' developmental trajectories (e.g., caste fate) [39], [40], and within-group (e.g., within-colony) conflict between social partners has long been recognized by students of social evolution to have widespread evolutionary effects [49]–[51]. The balance of within-group selection and between-group selection—as famously described by Hamilton's Rule [46]–[48], [52], [53]—together with the underlying genetic architecture governs the coevolution of interacting social phenotypes [54], [55]. The complex array of interactions that characterize social insect colonies can be considered an emergent property of the social group and earlier models suggest that selection at the colony-level shapes these networks of interactions [56], [57]. Indeed, more recent theory and empirical studies demonstrate that selection at the group level (i.e. the benefit term in Hamilton's Rule) acts much more effectively than within-group selection (i.e. the cost term in Hamilton's Rule) on genetic components influencing social interactions [14], [54], [55], [58]–[60]. Thus, the genetic basis of complex social phenotypes may favor group-level evolutionary responses, leading to coadapted nestmate phenotypes that maximize group productivity [61].

Studies in other social insects, especially the fire ant *Solenopsis invicta* and the honey bee *Apis mellifera* have also found evidence for genetic effects that are dependent on the social context provided by nestmates [62]–[70]. Thus, intergenomic epistasis may be a common feature within and between social insect populations. Complex social interactions such as those found in social insect colonies may often give rise to intergenomic epistasis, affect evolutionary dynamics within populations and also contribute to phenotypic differences between lineages.

## Materials and Methods

### Origin of workers and brood

In September 2004, nests of *Temnothorax curvispinosus* were collected from Griffy Nature Preserve, Bloomington, IN, USA, and nests of *T. longispinosus* were collected from two sites, Rondeau Provincial Park, ON, Canada and Allegany State Park, NY, USA. These nests were overwintered in the laboratory [29], [71]. Nests of overwintered *T. ambiguus* were collected on 16 April 2005 from Cowling Arboretum, Northfield, MN, USA.

### Experimental design

During the last two weeks of April 2005, I created experimental colonies using workers and larvae from the overwintered colonies. Pools of workers and larvae from all colonies of a single species (or collection site in the case of *T. longispinosus*) were used to create a total of 15 replicate experimental colonies, each with 15 workers and 25 larvae, for each of the nine worker-brood species combination. Pools of workers and larvae were used to minimize the contribution of variation between field-collected nests to variation between replicate experimental colonies.

Replicate experimental colonies were kept in climate control chambers simulating seasonal conditions [29], [71]. Water and freshly frozen adult fruit flies were provided *ad libitum* and refreshed weekly, and 1.5 ml of 10% sucrose solution was provided to each replicate colony at the beginning of the experiment. Colonies were checked biweekly and new worker pupae were removed, frozen, and weighed to the nearest 0.001 mg with a Sartorius MC-5 microbalance (Sartorius, Edgewood, NY). New workers were removed as pupae and not as adults to ensure that no old workers were mistaken for new workers. The first worker pupae were removed during the last week of June and most had been removed by the last week of July 2005. Wet mass, which is strongly correlated with dry mass, was used as a measurement of body size [29]. At the end of the study, old field-collected workers from each worker-brood combinations were weighed to provide an estimate of natural interspecific size variation.

### Statistical analysis

Phenotypic data were analyzed with the following model using the mixed model procedure of SAS:
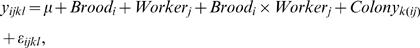
where *y_ijkl_* is the observed mass of a new worker focal individual; µ is the overall mean; *Brood_i_* is the species identity of brood, i.e. focal individuals, that develop into new workers (fixed effect); *Worker_j_* is the species identity of care-giving workers (fixed effect); *Brood_i_×Worker_j_* is the interaction between brood and worker species (fixed effect); *Colony_k(ij)_* is replicate colony nested within brood and worker species (random effect); ε*_ijkl_* is random error (*4, 5*). While *T. longispinosus* nests were collected from two sites, there was no difference in the mass of old care-giving *T. longispinosus* workers between the two collection sites (general linear model, *F*_(1,307)_ = 0.17, *P* = 0.68), and there was also no difference in brood effect (mixed model, *F*_(1,263)_ = 1.46, *P* = 0.23) or worker effect (*F*_(1,411)_ = 0.36, *P* = 0.55) for new worker mass for the two collection sites. Thus, only species identity was considered. A model including heterogeneous error variances was used [72] because error variances were not homogeneous across worker-brood combinations (Levene's test, *P*<0.01). Fisher's post-hoc test was used to compare different worker-brood combinations. To test whether there was differential survival (as indicated by the number of workers remaining at the end of the study) among the three old worker species, I used a generalized linear model with Poisson distributed residuals and a log link function. To control for a correlation between new worker mass and the total number of individuals produced across colonies, I performed a separate analysis using residuals from the regression of total colony production on mean new worker mass. For mixed model analyses and associated post-hoc tests I used SAS, and for the remaining analyses I used Statistica 6.1 software.
